# Supplementary material for: The impact of new urbanization on water ecological resilience: An empirical study from central China
Source: PLoS One. 2024 Dec 9;19(12):e0313865. doi: 10.1371/journal.pone.0313865 (PMC11627381; doi:10.1371/journal.pone.0313865)
Supplement: Attachment — Submitted filename: Review comments.pdf [file pone.0313865.s003.pdf]

The manuscript presents a comprehensive analysis of the relationship between new urbanization and water ecological resilience (WER) in central China. The study is timely and relevant, given the increasing global concern for sustainable urban development and the protection of water ecosystems. The authors have employed a robust methodology, utilizing various econometric models such as the STIRPAT model, dynamic panel models, and panel threshold effect models to test their hypotheses. The use of a quasi-natural experiment based on comprehensive pilot projects for new urbanization adds a valuable layer to the analysis.

The manuscript is well-structured, with a clear abstract, introduction, literature review, methodology, results, and conclusion. The arguments are well-supported by empirical evidence, and the data analysis is thorough. The authors have also addressed potential endogeneity issues through the use of system GMM estimation, which strengthens the reliability of their findings.

In terms of minor suggestions for improvement:

1. The authors may consider providing a more detailed discussion on the policy implications of their findings, particularly how the results can inform future urban planning and water resource management strategies in central China.

2. It would be beneficial to include a comparison of the study's findings with other regional or global studies to contextualize the results within a broader framework.

Overall, the manuscript is of high quality and makes a significant contribution to the field of urbanization and environmental sustainability. I have no reservations in recommending this paper for acceptance in PLOS ONE.
