# Supplementary material for: The impact of new urbanization on water ecological resilience: An empirical study from central China
Source: PLoS One. 2024 Dec 9;19(12):e0313865. doi: 10.1371/journal.pone.0313865 (PMC11627381; doi:10.1371/journal.pone.0313865)
Supplement: S1 File — (ZIP) [file pone.0313865.s002.zip › Table 1-12.zip!/Table 1.docx]

Table 1. Index system of *NU*

| **Target Layer** | **Criterion Layer** | **Indicator Layer** |
| --- | --- | --- |
| **New Urbanization** | Population Urbanization | Number of registered unemployed persons in urban areas (persons)  Permanent resident urbanization rate (%)  Proportion of employees in secondary industry (%)  Proportion of employees in tertiary industry (%) |
|  | Economic Urbanization | GDP per capita (Yuan)  Total investment in fixed assets (10^8^ Yuan)  Proportion of non-agricultural output value (%)  Per capita disposable income of urban residents (%)  Per capita actual utilization of foreign investment (Yuan) |
|  | Spatial Urbanization | Proportion of urban construction land in urban area (%)  Park green area (hm^2^)  Built-up area (km^2^)  Per capita urban road area (m^2^)  Green coverage rate of built-up area (%)  Green area ratio (%) |
|  | Social Urbanization | Total retail sales of consumer goods (10^4^ Yuan)  Number of museums (number)  Number of Internet broadband access users (10^4^ households)  Number of hospital beds (sheets)  Urban gas penetration rate (%)  Municipal expenditure on science and technology (10^4^ Yuan)  Education expenditure of the city (10^4^ Yuan) |
