# Supplementary material for: The impact of new urbanization on water ecological resilience: An empirical study from central China
Source: PLoS One. 2024 Dec 9;19(12):e0313865. doi: 10.1371/journal.pone.0313865 (PMC11627381; doi:10.1371/journal.pone.0313865)
Supplement: S1 File — (ZIP) [file pone.0313865.s002.zip › Table 1-12.zip!/Table 10.docx]

Table 10. Impact of *NU* on intermediary variables (regions with higher levels of *NU*)

| **Variable** | **ln*Ec*** | **ln*Pe*** | **ln*In*** | **ln*Ft*** | **ln*Fs*** | **ln*Ts*** | **ln*Hu*** | **ln*Ms*** | **ln*Ma*** |
| --- | --- | --- | --- | --- | --- | --- | --- | --- | --- |
| Constant term | 3.048^*^  （0.069） | 3.779^*^  （0.090） | 3.724^*^  （0.076） | 3.767^*^  （0.061） | 3.659^**^  （0.034） | 3.593^*^  （0.055） | 3.528^*^  （0.052） | 3.296^**^  （0.030） | 3.700^**^  （0.035） |
| Dependent variable lagged one period | 0.256^*^  （0.085） | 0.271^**^  （0.026） | 0.270^**^  （0.033） | 0.275^*^  （0.054） | 0.268^*^  （0.067） | 0.274^*^  （0.062） | 0.293^**^  （0.041） | 0.259^*^  （0.074） | 0.294^*^  （0.052） |
| ln*NU* | 0.054^**^  （0.037） | 0.038^**^  （0.043） | 0.046^*^  （0.075） | 0.027^**^  （0.018） | 0.092^**^  （0.026） | 0.089^*^  （0.057） | 0.134^*^  （0.089） | 0.148^*^  （0.053） | 0.125^*^  （0.076） |
| Control variable | YES | YES | YES | YES | YES | YES | YES | YES | YES |
| Arellano-Bond AR（1） | -2.907  （0.003） | -2.223  （0.011） | -2.651  （0.007） | -3.524  （0.000） | -3.748  （0.000） | -3.045  （0.002） | -2.770  （0.005） | -2.653  （0.006） | -2.326  （0.009） |
| Arellano-Bond AR（2） | 0.927  （0.355） | 1.545  （0.206） | 1.362  （0.259） | 0.596  （0.517） | 0.467  （0.551） | 0.900  （0.374） | 1.333  （0.272） | 1.364  （0.261） | 1.491  （0.215） |
| Sargan test | 26.741  （0.360） | 20.683  （0.635） | 23.604  （0.582） | 28.906  （0.241） | 29.909  （0.218） | 27.511  （0.343） | 26.007  （0.396） | 24.235  （0.587） | 22.192  （0.604） |

Note: ^*^, ^**^, and ^***^ indicate that the variable is significant at the level of 10%, 5%, and 1%, respectively.
