# Supplementary material for: The impact of new urbanization on water ecological resilience: An empirical study from central China
Source: PLoS One. 2024 Dec 9;19(12):e0313865. doi: 10.1371/journal.pone.0313865 (PMC11627381; doi:10.1371/journal.pone.0313865)
Supplement: S1 File — (ZIP) [file pone.0313865.s002.zip › Table 1-12.zip!/Table 11.docx]

Table 11. Results of difference-in-difference estimation

| **Variable** | **Model 1** | **Model 2** | **Model 3** | **Model 4** |
| --- | --- | --- | --- | --- |
| Constant term | 3.523^*^  （0.084） | 2.190^**^  （0.038） | 2.435^*^  （0.096） | 3.127^*^  （0.051） |
| ln*WER*_t-1_ | 0.261^*^  （0.078） | 0.307^*^  （0.052） | 0.284^*^  （0.080） | 0.315^**^  （0.046） |
| ln*NU*Zh*TP* | 0.347^***^  （0.006） | 0.249^**^  （0.025） | 0.308^***^  （0.002） | 0.192^*^  （0.083） |
| Control variable |  |  | YES | YES |
| Individual fixed effect |  | YES |  | YES |
| Time fixed effect |  | YES |  | YES |
| *R^2^* | 0.519 | 0.593 | 0.781 | 0.870 |
| *F* | 62.335^***^ | 54.926^***^ | 45.687^***^ | 40.254^***^ |

Note: ^*^, ^**^, and ^***^ indicate that the variable is significant at the level of 10%, 5%, and 1%, respectively.
