# Supplementary material for: The impact of new urbanization on water ecological resilience: An empirical study from central China
Source: PLoS One. 2024 Dec 9;19(12):e0313865. doi: 10.1371/journal.pone.0313865 (PMC11627381; doi:10.1371/journal.pone.0313865)
Supplement: S1 File — (ZIP) [file pone.0313865.s002.zip › Table 1-12.zip!/Table 12.docx]

Table 12. Estimated results of robustness test

| **Variable** | **PSM-DID** | **Consider other policies** |
| --- | --- | --- |
| Constant term | 3.348^**^  （0.026） | 2.902^*^  （0.074） |
| ln*WER*_t-1_ | 0.293^*^  （0.069） | 0.271^**^  （0.035） |
| ln*NU*Zh*TP* | 0.185^**^  （0.042） | 0.178^*^  （0.057） |
| Control variable | YES | YES |
| Individual fixed effect | YES | YES |
| Time fixed effect | YES | YES |
| *R^2^* | 0.847 | 0.853 |
| *F* | 42.001^***^ | 39.526^***^ |

Note: ^*^, ^**^, and ^***^ indicate that the variable is significant at the level of 10%, 5%, and 1%, respectively.
