# Supplementary material for: The impact of new urbanization on water ecological resilience: An empirical study from central China
Source: PLoS One. 2024 Dec 9;19(12):e0313865. doi: 10.1371/journal.pone.0313865 (PMC11627381; doi:10.1371/journal.pone.0313865)
Supplement: S1 File — (ZIP) [file pone.0313865.s002.zip › Table 1-12.zip!/Table 2.docx]

Table 2. Index system of *WER*

| **Target Layer** | **Criterion Layer** | **Indicator Layer** |
| --- | --- | --- |
| **Water Ecological Resilience** | Driving Force | Urban population density (people /km^2^)  GDP Growth rate (%)  Natural population growth rate (%) |
|  | Pressure | Sewage discharge (10^4^ m^3^)  Population using water (10^4^ persons)  Water consumption of urban residents (10^8^ m^3^)  Total industrial water consumption (10^8^ m^3^)  Urban public water consumption (10^8^ m^3^)  Industrial wastewater discharge (10^4^ tons)  Leakage of public water supply (10^4^ m^3^) |
|  | State | Total water resources (10^8^ m^3^)  Total water supply (10^8^ m^3^)  Per capita water supply (m^3^)  Water access rate (%)  Forest coverage (%)  Proportion of wetland area to national land area (%) |
|  | Influence | Industrial water satisfaction (%)  Agricultural water satisfaction (%)  Domestic water satisfaction (%)  Proportion of soil erosion area (%) |
|  | Response | Urban sewage treatment rate (%)  Total sewage treatment (10^4^ tons)  Sewage treatment plant sewage treatment capacity (10^4^ m^3^/ day)  Industrial water reuse (10^4^ m^3^)  Number of wastewater treatment facilities (sets)  Drainage pipe density in built-up area (km/km^2^)  Soil erosion control rate (%)  Proportion of water-saving irrigation area (%)  Proportion of environmental pollution control investment in GDP (%) |
