# Supplementary material for: The impact of new urbanization on water ecological resilience: An empirical study from central China
Source: PLoS One. 2024 Dec 9;19(12):e0313865. doi: 10.1371/journal.pone.0313865 (PMC11627381; doi:10.1371/journal.pone.0313865)
Supplement: S1 File — (ZIP) [file pone.0313865.s002.zip › Table 1-12.zip!/Table 3.docx]

Table 3. Descriptive statistical results

| **Variable** | **ln*WER*** | **ln*NU*** | **ln*A*** | **ln*T*** | **ln*Wb*** | **ln*Qi*** | **ln*Ec*** | **ln*Pe*** | **ln*In*** | **ln*Ft*** | **ln*Fs*** | **ln*Ts*** | **ln*Hu*** | **ln*Ms*** | **ln*Ma*** |
| --- | --- | --- | --- | --- | --- | --- | --- | --- | --- | --- | --- | --- | --- | --- | --- |
| Mean | -0.577 | -2.407 | 9.382 | 7.371 | 7.347 | 6.932 | 6.435 | 6.094 | -0.689 | -0.895 | 1.616 | 1.142 | 2.138 | -0.271 | 1.772 |
| Median | -0.546 | -2.303 | 9.210 | 7.388 | 7.276 | 6.875 | 6.227 | 6.020 | -0.636 | -0.884 | 1.535 | 1.151 | 2.149 | -0.282 | 1.767 |
| Maximum | -0.134 | -1.138 | 10.839 | 11.367 | 8.464 | 7.526 | 9.782 | 7.219 | 0.000 | -0.109 | 2.001 | 2.708 | 2.545 | -0.016 | 2.281 |
| Minimum | -0.853 | -4.269 | 7.894 | 3.296 | 5.553 | 4.042 | 1.411 | 4.205 | -2.577 | -2.262 | 0.794 | -0.476 | 1.907 | -0.603 | 1.194 |
