# Supplementary material for: The impact of new urbanization on water ecological resilience: An empirical study from central China
Source: PLoS One. 2024 Dec 9;19(12):e0313865. doi: 10.1371/journal.pone.0313865 (PMC11627381; doi:10.1371/journal.pone.0313865)
Supplement: S1 File — (ZIP) [file pone.0313865.s002.zip › Table 1-12.zip!/Table 4.docx]

Table 4. Estimation results of dynamic panel model

| **Variable** | **OLS** | **FE** | **DIF-GMM** | **SYS-GMM** |
| --- | --- | --- | --- | --- |
| Constant term | 1.168  （0.185） | 1.217  （0.256） | 0.634^*^  （0.071） | 0.517^*^  （0.073） |
| ln*WER*_t-1_ | 0.332  （0.207） | 0.382^**^  （0.031） | 0.298^*^  （0.083） | 0.329^*^  （0.090） |
| ln*NU* | -0.211^**^  （0.026） | -0.237^**^  （0.043） | -0.197^**^  （0.015） | -0.161^**^  （0.034） |
| ln*NU*^2^ | 0.183^***^  （0.005） | 0.136^**^  （0.014） | 0.152^**^  （0.030） | 0.115^**^  （0.026） |
| ln*A* | -0.154^**^  （0.019） | -0.135^**^  （0.023） | -0.096^**^  （0.047） | -0.103^**^  （0.014） |
| ln*T* | 0.193^*^  （0.087） | 0.159^**^  （0.046） | 0.245^*^  （0.072） | 0.218^*^  （0.095） |
| ln*Wb* | 0.149^**^  （0.040） | 0.160^*^  （0.068） | 0.129^***^  （0.003） | 0.086^***^  （0.007） |
| ln*Qi* | 0.127^**^  （0.034） | 0.094^**^  （0.029） | 0.088^*^  （0.054） | 0.052^*^  （0.081） |
| Individual fixed effect | YES | YES | YES | YES |
| Time fixed effect | YES | YES | YES | YES |
| *R*^2^ | 0.926 | 0.785 |  |  |
| Arellano-Bond AR（1） |  |  | -2.542  （0.011） | -2.714  （0.007） |
| Arellano-Bond AR（2） |  |  | 0.785  （0.392） | 1.273  （0.286） |
| Sargan test |  |  | 9.003  （0.068） | 28.158  （0.397） |

Note: ^*^, ^**^, and ^***^ indicate that the variable is significant at the level of 10%, 5%, and 1%, respectively.
