# Supplementary material for: The impact of new urbanization on water ecological resilience: An empirical study from central China
Source: PLoS One. 2024 Dec 9;19(12):e0313865. doi: 10.1371/journal.pone.0313865 (PMC11627381; doi:10.1371/journal.pone.0313865)
Supplement: S1 File — (ZIP) [file pone.0313865.s002.zip › Table 1-12.zip!/Table 5.docx]

Table 5. Estimated results of robustness test

| **Variable** | **Extreme value processing** | | **Re-measure the independent variable** | | **Replace the model** |
| --- | --- | --- | --- | --- | --- |
|  | **(1)** | **(2)** | **(3)** | **(4)** | **(5)** |
| Constant term | 0.648^*^  （0.057） | 0.579^*^  （0.063） | 0.724^*^  （0.078） | 0.589^*^  （0.075） | 1.499^**^  （0.021） |
| ln*WER*_t-1_ | 0.319^**^  （0.041） | 0.304^*^  （0.086） | 0.286^*^  （0.093） | 0.297^**^  （0.036） | 0.278^*^  （0.064） |
| ln*NU* | -0.156^***^  （0.009） | -0.150^**^  （0.037） | -0.169^**^  （0.045） | -0.151^**^  （0.028） | -0.185^*^  （0.073） |
| ln*NU*^2^ | 0.113^*^  （0.065） | 0.121^**^  （0.018） | 0.127^*^  （0.072） | 0.110^**^  （0.034） | 0.134^**^  （0.047） |
| Control variable | YES | YES | YES | YES | YES |
| Individual fixed effect | YES | YES | YES | YES | YES |
| Time fixed effect | YES | YES | YES | YES | YES |
| Arellano-Bond AR（1） | -2.792  （0.006） | -3.895  （0.000） | -4.016  （0.000） | -2.803  （0.005） |  |
| Arellano-Bond AR（2） | 1.286  （0.279） | 0.587  （0.541） | 0.809  （0.407） | 1.264  （0.291） |  |
| Sargan test | 25.653  （0.571） | 30.294  （0.242） | 29.362  （0.314） | 29.007  （0.386） |  |

Note: ^*^, ^**^, and ^***^ indicate that the variable is significant at the level of 10%, 5%, and 1%, respectively.
