# Supplementary material for: The impact of new urbanization on water ecological resilience: An empirical study from central China
Source: PLoS One. 2024 Dec 9;19(12):e0313865. doi: 10.1371/journal.pone.0313865 (PMC11627381; doi:10.1371/journal.pone.0313865)
Supplement: S1 File — (ZIP) [file pone.0313865.s002.zip › Table 1-12.zip!/Table 6.docx]

Table 6. Threshold effect test

| **Threshold variable** | **Threshold number** | **Threshold value** | **F-value** | **P-value** | **Critical value** | | | **Number of BS** |
| --- | --- | --- | --- | --- | --- | --- | --- | --- |
|  |  |  |  |  | **10%** | **5%** | **1%** |  |
| ln*NU* | Single threshold | -0.361 | 16.27 | 0.049 | 14.228 | 16.245 | 21.316 | 400 |
|  | Double threshold | 1.613 | 6.12 | 0.637 | 20.101 | 24.359 | 29.628 | 400 |
