# Supplementary material for: The impact of new urbanization on water ecological resilience: An empirical study from central China
Source: PLoS One. 2024 Dec 9;19(12):e0313865. doi: 10.1371/journal.pone.0313865 (PMC11627381; doi:10.1371/journal.pone.0313865)
Supplement: S1 File — (ZIP) [file pone.0313865.s002.zip › Table 1-12.zip!/Table 7.docx]

Table 7. Regression results of threshold effect

| **Variable** | | **Coefficient estimates** | **P-value** |
| --- | --- | --- | --- |
| 95% confidence interval | ln*NU*≤-0.361（*NU*≤0.697） | -0.209^*^ | 0.076 |
| [-0.368, -0.196] | ln*NU>*-0.361（*NU*>0.697） | 0.156^**^ | 0.043 |
| Constant term | | 3.507^**^ | 0.039 |
| Control variable | | YES | |
| Individual fixed effect | | YES | |
| Time fixed effect | | YES | |
| *R^2^* | | 0.875 | |

Note: ^*^, ^**^, and ^***^ indicate that the variable is significant at the level of 10%, 5%, and 1%, respectively.
