# Supplementary material for: The impact of new urbanization on water ecological resilience: An empirical study from central China
Source: PLoS One. 2024 Dec 9;19(12):e0313865. doi: 10.1371/journal.pone.0313865 (PMC11627381; doi:10.1371/journal.pone.0313865)
Supplement: S1 File — (ZIP) [file pone.0313865.s002.zip › Table 1-12.zip!/Table 8.docx]

Table 8. Impact of *NU* and intermediary variables on *WER*

| **Variable** | **Regions with lower levels of *NU*** | | **Regions with higher levels of *NU*** | |
| --- | --- | --- | --- | --- |
|  | **Model 1** | **Model 2** | **Model 3** | **Model 4** |
| Constant term | 1.964^*^  （0.079） | 2.239^*^  （0.056） | 3.005^**^  （0.037） | 2.412^*^  （0.074） |
| ln*WER*_t-1_ | 0.352^**^  （0.024） | 0.201^*^  （0.075） | 0.343^*^  （0.068） | 0.228^*^  （0.057） |
| ln*NU* | -0.267^**^  （0.036） | -0.183^**^  （0.040） | 0.246^***^  （0.009） | 0.175^*^  （0.081） |
| ln*Ec* |  | -0.169^***^  （0.004） |  | -0.072^**^  （0.036） |
| ln*Pe* |  | -0.137^*^  （0.058） |  | -0.069^**^  （0.043） |
| ln*In* |  | -0.105^*^  （0.070） |  | -0.048^*^  （0.094） |
| ln*Ft* |  | -0.124^**^  （0.036） |  | -0.066^*^  （0.051） |
| ln*Fs* |  | 0.048^*^  （0.061） |  | 0.107^*^  （0.065） |
| ln*Ts* |  | 0.052^**^  （0.037） |  | 0.139^**^  （0.023） |
| ln*Hu* |  | 0.090  （0.126） |  | 0.112^*^  （0.074） |
| ln*Ms* |  | 0.049^*^  （0.078） |  | 0.081^*^  （0.057） |
| ln*Ma* |  | 0.083  （0.215） |  | 0.096^*^  （0.063） |
| Control variable | YES | YES | YES | YES |
| Arellano-Bond AR（1） | -3.711  （0.000） | -2.924  （0.004） | -2.450  （0.008） | -3.204  （0.002） |
| Arellano-Bond AR（2） | 0.563  （0.558） | 1.259  （0.295） | 1.416  （0.232） | 0.850  （0.401） |
| Sargan test | 31.405  （0.239） | 28.261  （0.388） | 24.115  （0.593） | 29.895  （0.336） |

Note: ^*^, ^**^, and ^***^ indicate that the variable is significant at the level of 10%, 5%, and 1%, respectively.
