# Supplementary material for: The impact of new urbanization on water ecological resilience: An empirical study from central China
Source: PLoS One. 2024 Dec 9;19(12):e0313865. doi: 10.1371/journal.pone.0313865 (PMC11627381; doi:10.1371/journal.pone.0313865)
Supplement: S1 File — (ZIP) [file pone.0313865.s002.zip › Table 1-12.zip!/Table 9.docx]

Table 9. Impact of *NU* on intermediary variables (regions with lower levels of *NU*)

| **Variable** | **ln*Ec*** | **ln*Pe*** | **ln*In*** | **ln*Ft*** | **ln*Fs*** | **ln*Ts*** | **ln*Hu*** | **ln*Ms*** | **ln*Ma*** |
| --- | --- | --- | --- | --- | --- | --- | --- | --- | --- |
| Constant term | 3.868^**^  （0.036） | 3.982^*^  （0.064） | 3.937^*^  （0.080） | 3.292^*^  （0.075） | 4.005^**^  （0.042） | 4.081^*^  （0.097） | 3.607^*^  （0.053） | 3.370^**^  （0.031） | 3.674^*^  （0.056） |
| Dependent variable lagged one period | 0.283^*^  （0.071） | 0.291^*^  （0.053） | 0.282^**^  （0.035） | 0.269^*^  （0.094） | 0.287^*^  （0.056） | 0.264^**^  （0.028） | 0.276^**^  （0.042） | 0.265^*^  （0.078） | 0.280^*^  （0.067） |
| ln*NU* | 0.168^**^  （0.029） | 0.187^**^  （0.016） | 0.154^*^  （0.079） | 0.115^**^  （0.040） | 0.052^*^  （0.083） | 0.039  （0.252） | 0.065  （0.190） | 0.049^*^  （0.054） | 0.030  （0.161） |
| Control variable | YES | YES | YES | YES | YES | YES | YES | YES | YES |
| Arellano-Bond  AR（1） | -2.756  （0.007） | -3.895  （0.000） | -3.163  （0.002） | -2.418  （0.009） | -2.886  （0.004） | -3.663  （0.000） | -3.021  （0.003） | -2.312  （0.010） | -2.759  （0.005） |
| Arellano-Bond  AR（2） | 1.335  （0.264） | 0.457  （0.561） | 0.882  （0.379） | 1.470  （0.217） | 1.307  （0.280） | 0.584  （0.528） | 0.909  （0.362） | 1.514  （0.216） | 1.333  （0.267） |
| Sargan test | 25.112  （0.577） | 31.818  （0.216） | 29.267  （0.335） | 23.609  （0.598） | 27.668  （0.392） | 30.745  （0.241） | 28.447  （0.356） | 22.003  （0.629） | 25.786  （0.580） |

Note: ^*^, ^**^, and ^***^ indicate that the variable is significant at the level of 10%, 5%, and 1%, respectively.
