# Supplementary material for: The impact of new urbanization on water ecological resilience: An empirical study from central China
Source: PLoS One. 2024 Dec 9;19(12):e0313865. doi: 10.1371/journal.pone.0313865 (PMC11627381; doi:10.1371/journal.pone.0313865)
Supplement: S1 File — (ZIP) [file pone.0313865.s002.zip › Fig 1. Map of the central China.zip!/Fig 1.docx]

Fig 1. Map of the central China

Note: This map was created based on the standard map service website of the State Administration of Surveying, Mapping and Geoinformation, which downloaded the standard map with the review number GS(2019)1822. The base map has not been modified.
