# Supplementary material for: The impact of new urbanization on water ecological resilience: An empirical study from central China
Source: PLoS One. 2024 Dec 9;19(12):e0313865. doi: 10.1371/journal.pone.0313865 (PMC11627381; doi:10.1371/journal.pone.0313865)
Supplement: Attachment — Submitted filename: Response to Reviewers.docx [file pone.0313865.s004.docx]

Author's Reply to the Review Report1

We sincerely appreciate all valuable comments and suggestions, which helped us to improve the quality of the manuscript.

1. The authors may consider providing a more detailed discussion on the policy implications of their findings, particularly how the results can inform future urban planning and water resource management strategies in central China.

In response to the reviewer’s comment, we revised the conclusions and policy recommendations in lines 684-692 and 698-705 as follows: (1)At the same time, it is essential to comprehensively implement the strategy of ecological civilization, effectively and solidly advance the construction of water ecological civilization, integrate the management of water resources, water environment, and water ecology, timely enhance regulations on the water ecological environment, improve the efficiency of regulatory implementation, and strive to elevate the standards of water ecological protection. This approach will ensure that while pursuing NU development, we effectively promote the restoration and enhancement of WER and achieve harmonious coexistence and coordinated development between humanity and nature. (2)Therefore, for regions with lower levels of NU, it is essential to not only strengthen NU construction and focus on improving its quality and efficiency but also actively transform the mode of economic growth by improving comprehensive resource utilization and facilitating the development of a circular economy. Additionally, it is important to enhance population structure through refined fertility policies and population mobility strategies, optimize investment structures by increasing investments in new energy infrastructure and digital infrastructure, and adjust the foreign trade development model by leveraging digital technologies to promote digital trade.

2. It would be beneficial to include a comparison of the study's findings with other regional or global studies to contextualize the results within a broader framework.

In response to the reviewer’s comment, we revised analysis of empirical results in lines 455-467 and the conclusions and policy recommendations in lines 729-734 as follows: (1)This result is similar to the study by He et al. (2024) and Zhao and Luo (2024). The former found that the coupling and coordination degree of urbanization and ecological resilience in the Three Gorges Reservoir Area remained basically unchanged, mainly at the stage of “slight imbalance”, “impending imbalance”, “reluctant coordinated” or “intermediate coordination”, most of which showed uncoordinated types of lagging ecological resilience or being hindered from urbanization. The latter found that, from the perspective of coupling and coordination, the grinding effect between the urbanization system and the ecological environment system in China at city level continues to be good, the number of cities with mild or lower levels of dysregulation and coordination development types is constantly decreasing. Overall, China has stepped into the stage of transformation and development. However, there is an emerging trend towards lagging ecological environments, with varying spatial distribution from south to north. (2)The findings of this study can enhance the level of WER in the promotion of NU within central China. Furthermore, these results serve as a reference for other developing countries and regions akin to central China, aiming to elevate their WER during the advancement of NU. This paper improves the analysis of the impact of NU on WER and enriches the theory of NU and ecological resilience. The econometric model and empirical methodology employed in this study can be applied on a global scale.

Author's Reply to the Review Report2

We sincerely appreciate all valuable comments and suggestions, which helped us to improve the quality of the manuscript.

1. In general groundwater contains large freshwater reserves than surface water, but the authors given contradictory statement such as “In 2021, the total water resources in central China were 617.95 billion m^3^ including 586.54 billion m^3^ of surface water and 167.8billion m^3^ of groundwater reserves. Check once.

In response to the reviewer’s comment, we once again consulted the statistical yearbook to ensure that the data was accurate. We also checked relevant data from China, as follows: In 2022, the total water resources in China were 27088.1 billion m^3^ including 25984.4 billion m^3^ of surface water and 7924.4 billion m^3^ of groundwater reserves.

2. Legend in Figure 1 is not visible. Increase the font size.

In response to the reviewer’s comment, we revised Figure 1.

3. Recheck the equations.

In response to the reviewer’s comment, we rechecked the equations.

4. Is it typographical error “STIRPAT model---a stochastic” or it is correct expression. Check once.

In response to the reviewer’s comment, we revised the model construction in lines 287-289 as follows: Consequently, researchers have proposed an alternative methodology referred to as the STIRPAT model—a stochastic framework for assessing environmental impacts, specifically designed to capture the intricate dynamics underlying environmental influences.

5. There were several studies on STIRPAT model, emphasize this model among other models and justify selecting this model in introduction part and introduction shall end by aim of the study.

In response to the reviewer’s comment, we revised the introduction in lines 72-85 as follows: Currently, the academic community mainly uses the Kaya Identity, the LMDI model, and the traditional STIRPAT model to study the influencing factors of ecological environments. However, the individual factors in the decomposition of the Kaya Identity equation need to be analyzed annually or by time periods, and are subject to the constraint of maintaining an equal relationship. The LMDI model cannot examine the elasticity of each factor, while the traditional STIRPAT model only considers three aspects of population scale, wealth level and technical level, and cannot fully describe the impact of social and economic factors on ecological environments. In contrast, the STIRPAT model allows the impact of each factor to be estimated as a parameter, enabling researchers to extend the model according to their research objectives. Therefore, in order to achieve the research objectives of this paper, we use the extended STIRPAT model to analyze the impact of NU on WER in the central China, thereby providing scientific basis for formulating policies to enhance WER in the central China and other similar regions of the world.

6. Is the proposed methodology can be adopted globally or it has any limitations? It shall be clearly reflected in the manuscript.

In response to the reviewer’s comment, we revised the conclusions and policy recommendations in lines 729-734 as follows: The findings of this study can enhance the level of WER in the promotion of NU within central China. Furthermore, these results serve as a reference for other developing countries and regions akin to central China, aiming to elevate their WER during the advancement of NU. This paper improves the analysis of the impact of NU on WER and enriches the theory of NU and ecological resilience. The econometric model and empirical methodology employed in this study can be applied on a global scale.

7. Comparison with other studies shall enlighten the script to further extent.

In response to the reviewer’s comment, we revised the conclusions and policy recommendations in lines 684-692 and 698-705 as follows: (1)At the same time, it is essential to comprehensively implement the strategy of ecological civilization, effectively and solidly advance the construction of water ecological civilization, integrate the management of water resources, water environment, and water ecology, timely enhance regulations on the water ecological environment, improve the efficiency of regulatory implementation, and strive to elevate the standards of water ecological protection. This approach will ensure that while pursuing NU development, we effectively promote the restoration and enhancement of WER and achieve harmonious coexistence and coordinated development between humanity and nature. (2)Therefore, for regions with lower levels of NU, it is essential to not only strengthen NU construction and focus on improving its quality and efficiency but also actively transform the mode of economic growth by improving comprehensive resource utilization and facilitating the development of a circular economy. Additionally, it is important to enhance population structure through refined fertility policies and population mobility strategies, optimize investment structures by increasing investments in new energy infrastructure and digital infrastructure, and adjust the foreign trade development model by leveraging digital technologies to promote digital trade.

8. The authors state that “regions with low levels of NU in central China, the stress effect of NU on WER is more pronounced and regions with high levels of NU in central China, NU has a significantly positive impact on WER. IS NU stops at high level? What happens at intermediate level and what are the future implications? What is the current NU scenario in central China (high, low, or intermediate)? Shall reflect in the manuscript.

In response to the reviewer’s comment, we revised the Estimation results of dynamic panel mediation effect model in lines 544-602. The 80 prefecture-level cities in central China were categorized based on the threshold value of the *NU* level. Prefecture-level cities with *NU*≤0.697 were classified as regions with a lower *NU* level (approximately 76% of the sample size), while those with *NU*>0.697 were classified as regions with a higher *NU* level (approximately 24% of the sample size).
